# Curcumin, an Active Constituent of Turmeric Spice: Implication in the Prevention of Lung Injury Induced by Benzo(a) Pyrene (BaP) in Rats

**DOI:** 10.3390/molecules25030724

**Published:** 2020-02-07

**Authors:** Saleh A. Almatroodi, Faris Alrumaihi, Mohammed A. Alsahli, Mazen Fahad Alhommrani, Arif Khan, Arshad Husain Rahmani

**Affiliations:** 1 Department of Medical Laboratories, College of Applied Medical Sciences, Qassim University, Buraidah 52571, Saudi Arabia; smtrody@qu.edu.sa (S.A.A.); f_alrumaihi@qu.edu.sa (F.A.); shly@qu.edu.sa (M.A.A.); m.alhommrani@qu.edu.sa (M.F.A.); 2 Department of Basic Health Sciences, College of Applied Medical Science, Qassim University, Buraidah 52571, Saudi Arabia; 4140@qu.edu.sa

**Keywords:** benzo(a)pyrene, lung injury, inflammation, antioxidant, apoptosis

## Abstract

Benzo(a)pyrene (BaP) is a well-known carcinogen and enhances oxidative stress and apoptosis and also alters several molecular pathways. Curcumin is an active ingredient of *Curcuma longa*, and it has potent anti-inflammatory, antioxidant activity that defends cells from oxidative stress and cell death. The objectives of the present study were to explore the protective effects of curcumin against long-term administration of BaP induced disturbances in lungs of rats. Male rats were randomly divided into four groups: saline control, BaP only, BaP + curcumin, and curcumin only. Lung histopathology, electron microscopy, inflammatory cytokine release, antioxidant levels, apoptosis, and cell cycle were examined. Instillation of BaP significantly increased infiltration of inflammatory cells in alveolar space and inflammatory cytokine in blood. BaP induced lung tissue alterations including mild bronchitis, scant chronic inflammatory cell infiltrate in the wall of the respiratory bronchiole, and mild intra-alveolar haemorrhage. However, these alterations were found to be significantly less as mild inflammatory cell infiltrate in curcumin plus BaP treated group. Furthermore, electron microscopy results also showed necrotic changes and broken cell membrane of Type-II epithelial cell of alveoli in BaP group, which was reduced after adding curcumin treatment. In addition, we found BaP plus curcumin treatment effectively reduced inflammatory cytokines Tumour Necrosis Factor alpha (TNF-α), Interleukin 6 (IL-6), and C-reactive protein (CRP) levels in blood serum. Moreover, the levels of tunnel staining and p53 expression were significantly increased by BaP, whereas these changes were noticeably modulated after curcumin treatment. BaP also interferes in normal cell cycle, which was significantly improved with curcumin treatment. Overall, our findings suggest that curcumin attenuates BaP -induced lung injury, probably through inhibiting inflammation, oxidative stress and apoptosis in lung epithelial cells, and improving cell proliferation and antioxidants level. Thus, curcumin may be an alternative therapy for improving the outcomes of Benzo(a)pyrene-induced lung injury.

## 1. Introduction

Exposure to outdoor air pollution is connected to reduced lung function and play a vital role in the developing respiratory and diseases. In this context, Benzo(a)pyrene (BaP) is one of the potent carcinogen and certainly adsorbed on particles of diameters lower than 2 μm. Lung is one of the most susceptible organs to injuries/damage; being an interface between outer environment and body, it directly interacts with inhalant toxicants. Large pulmonary surface area and enormous vascular system further increase the vulnerability of the lungs [1,2]. Inhalation of BaP can directly induce inflammatory microenvironment in the lung or easily enter the lung alveoli and then such substances reach to different body organs via the vasculature [3,4]. A number of possible mechanisms have been proposed to explain these effects, comprising direct effects of particles that translocated into the systemic circulation and alterations of the cardiac and pulmonary and systemic oxidative stress and inflammatory responses that activate endothelial dysfunction, initiation of immune cells, and induction of cell death [5,6]. However, the exact mechanistic pathways are still not fully understood.

Several studies have revealed that exposure to benzopyrene causes endothelial disruption, DNA damage, impaired endogenous fibrinolysis, and altered lung function in human subjects [7,8]. Similarly, benzopyrene also plays a role in the alteration of surfactant dysfunction, endothelium-dependent vasodilatation, lipid metabolism, and modulates several signaling pathways and miRNAs in animal as well as in vitro studies [9,10,11]. Many toxicological studies demonstrated the severe adverse health effects of benzopyrene are linked with amplified oxidative stress and inflammation [5,12,13,14]. Thus, it is worthy to discover effective beneficial approaches for attenuating BaP associated ailments. Curcumin is the key isolated active ingredient of *Curcuma longa* Linn, which is broadly used as a spice as well as colouring agent in several herbal formulation and food preparation. Moreover, curcumin has been documented as an inhibitor of inflammation, oxidative stress and apoptosis in lung epithelial, as well as immune cells [15] and also plays role in the management of various cancers [16,17]. Certainly, curcumin treatment has proven to inhibit carcinogens induced lung injury as well as pathogenesis of various diseases including cancer.

Henceforth, an experimental study was performed to assess the toxicity of benzopyrene in rat lungs and evaluate the protective effects of curcumin against BaP induced oxidative stress, inflammation as well as cell death.

## 2. Results

### 2.1. Effects of Curcumin on Benzopyrene(BaP)-Induced Lung Tissue Alterations

**Figure 1:**
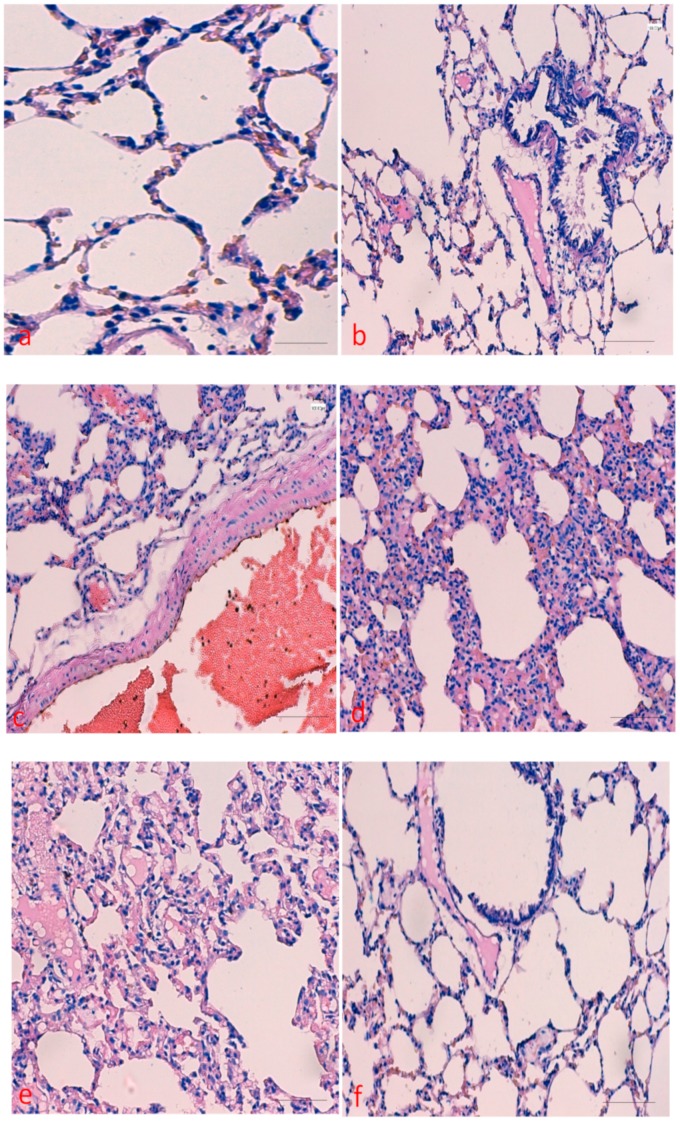
Effects of curcumin treatment on histopathological changes in BaP -induced lung tissue: Control group (**a**); BaP -induced group (**b**–**f**); (**b**) mild bronchitis, scant chronic inflammatory cell infiltrate in the wall of the respiratory bronchiole. Mild intra-alveolar haemorrhage as well, (**c**) moderate interstitial inflammatory cell infiltrate. Acute lung injury as intra-alveolar haemorrhage is also noted and acute on chronic changes; (**d**) moderate to marked mixed interstitial inflammatory cell infiltrate, suggestive of interstitial pneumonitis. Interstitial fibrosis is also appreciated; (**e**) interstitial chronic inflammatory cell infiltrate, suggestive of interstitial pneumonitis. Type 2 pneumocytes are also prominent; (**f**) hyper inflated alveolar spaces and mild septal inflammatory cell infiltrate. Scale bar = 50 μm.

To examine the protective effect of curcumin against BaP-induced lung injury, the rats were treated with doses of curcumin (50 mg/kg/bw). The lung tissues from all the experimental groups were analyzed through H&E staining and histological findings were compared accordingly. The results by haematoxylin and eosin staining confirmed that curcumin had protective role against BaP -induced lung damage. As shown in Figure 1a–e, BaP treated group showed mild bronchitis, scant chronic inflammatory cell infiltrate in the wall of the respiratory bronchiole and mild intra-alveolar haemorrhage as well and inflammatory cell infiltrate. Moderate to marked mixed interstitial inflammatory cell infiltrate, suggestive of interstitial pneumonitis. Interstitial fibrosis is also appreciated. In addition, Interstitial chronic inflammatory cell infiltrate, suggestive of interstitial pneumonitis. Type 2 pneumocytes are also prominent was seen. However, these alterations were found to be significantly less as mild inflammatory cell infiltrate in co-administration of curcumin plus BaP treated group (Figure 1f).

**Figure 2:**
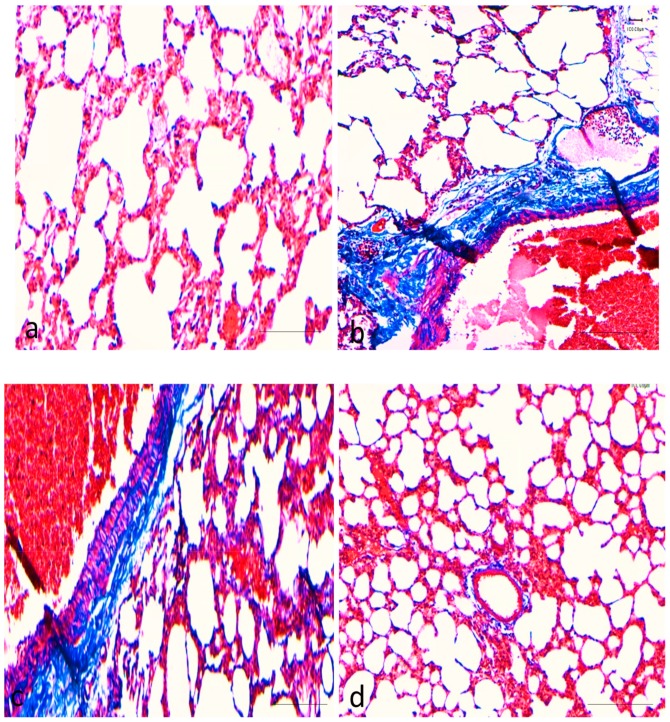
Effects of curcumin treatment on lung tissue: (**a**) control group: lung tissue structure was normal and no deposition of collagen fibre; (**b**) BaP -induced group showed extensive depositions of collagen fibre; (**c**) benzopyrene plus curcumin treated group: showed less depositions of collagen; (**d**) curcumin (50 mg/kg bw) group: there was no deposition of collagen fibre. Scale bar = 100 μm.

As shown in Figure 2a–d, BaP treated rat showed extensive depositions of collagen fibre, but group treated with BaP plus curcumin showed significantly less damage/ less depositions of collagen as evidence by Masson trichrome stain and sections stained blue staining. There was no collagen deposition was observed in the control group as well as the curcumin only treated group.

### 2.2. Effects of Curcumin on BaP-Induced Production of TNF-α, IL-6, and CRP

**Figure 3:**
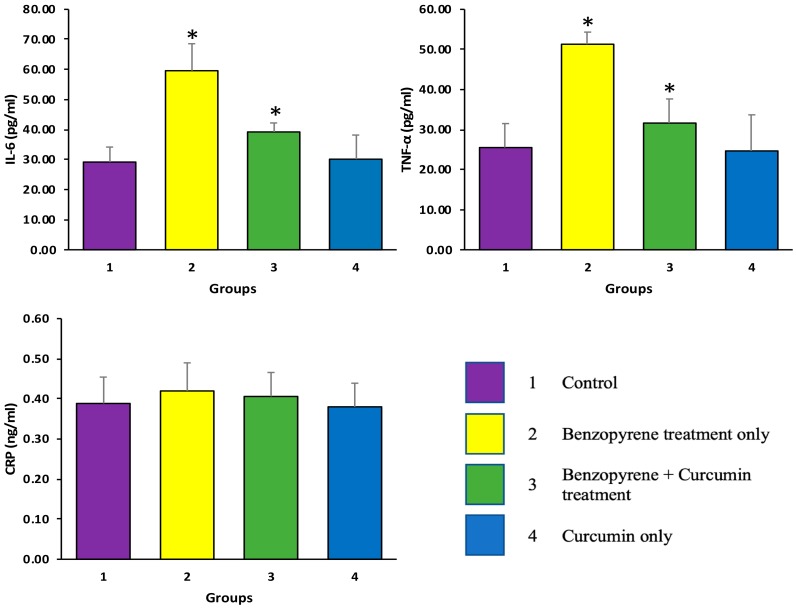
The anti-inflammatory effects of curcumin were evaluated through measurement of Tumour Necrosis Factor alpha (TNF-α), Interleukin 6 (IL-6), and C-reactive protein (CRP): BaP -induced group showed the levels of TNF-α and IL-6 were found to be substantial increased as compared with the control group (*p* <  0.05). However, production of TNF-α and IL-6 and were significantly decreased in the BaP plus curcumin treated group (*p* < 0.05). The level of CRP was increased in the BaP-treated group as compared to control group as well as co-administration of curcumin (50 mg/kg) with BaP (50 mg/kg), but difference was statically insignificant (*p* > 0.05).

Inflammation is implicated in the progression of pathogenesis of lung. Therefore, whether curcumin altered the levels of the inflammatory mediators in the experimental rats was observed. As shown in Figure 3a–d, ELISA based study revealed that the levels of TNF-α and IL-6 in the serum were significantly increased in the BaP-treated group compared with control group (*p* ≤ 0.05). In contrast, co-administration of curcumin (50 mg/kg) with BaP (50 mg/kg) exhibited significant reduction in TNF-α and the IL-6 level. Moreover, the level of CRP was increased in the BaP-treated group as compared to control group as well as co-administration of curcumin (50 mg/kg) with BaP (50 mg/kg), but the difference was statically insignificant (*p* > 0.05). These findings advocate that curcumin attenuates lung inflammation and decreased the inflammatory mediators, which might be induced by BaP in rats. The statistical comparison among groups was performed by one-way ANOVA.

### 2.3. Effect of Curcumin on Antioxidant Enzymes

**Figure 4:**
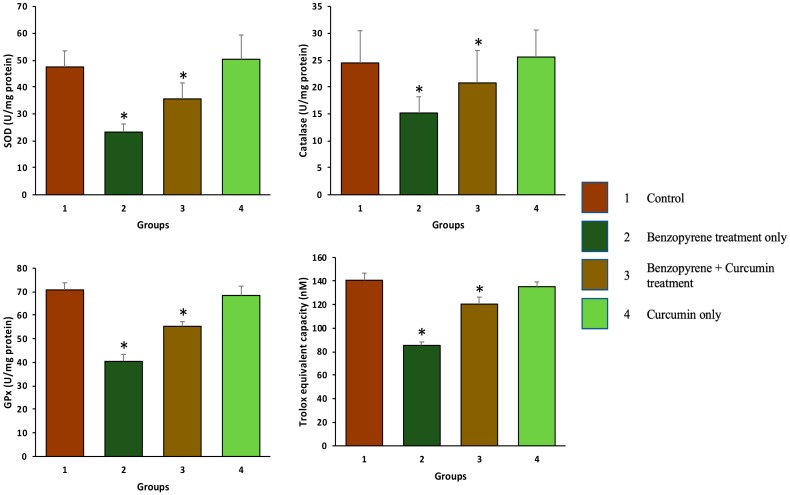
The antioxidant effects of curcumin were measured in the serum of different groups. Benzopyrene treated groups exhibited a decreased level of Glutathione peroxidase (GPx), superoxide dismutase (SOD), catalase (CAT), and total antioxidant capacity enzymes as compared to the control group (*p* < 0.05). Treatment of curcumin (50 mg/kg b.w.) in BaP administered rats increased the levels of SOD, CAT, GPx, and total antioxidant capacity significantly compared to the BaP treated group (*p* < 0.05).

Antioxidant levels in the serum of different group was measured to evaluate the role of curcumin as an antioxidant. Moreover, benzopyrene treated groups exhibited a decreased level of Glutathione peroxidase (GPx), superoxide dismutase (SOD), catalase (CAT) enzymes as compared to the control group (*p* < 0.05). Treatment of curcumin (50 mg/kg b.w.) in BaP administered rats increased the levels of SOD, CAT, and GPx significantly compared to BaP treated group (*p* < 0.05) (Figure 4).

### 2.4. Effect of Curcumin on Total Antioxidant Capacity (TAC)

Benzopyrene treated groups showed a low level of TAC as compared to the control group (*p* < 0.05). Treatment of curcumin (50 mg/kg b.w.) in BaP administered rats increased the levels of TAC significantly compared to BaP group (*p* < 0.05) (Figure 4).

### 2.5. Effect of Curcumin on Ultra-Structural Changes of Lung Tissue

**Figure 5:**
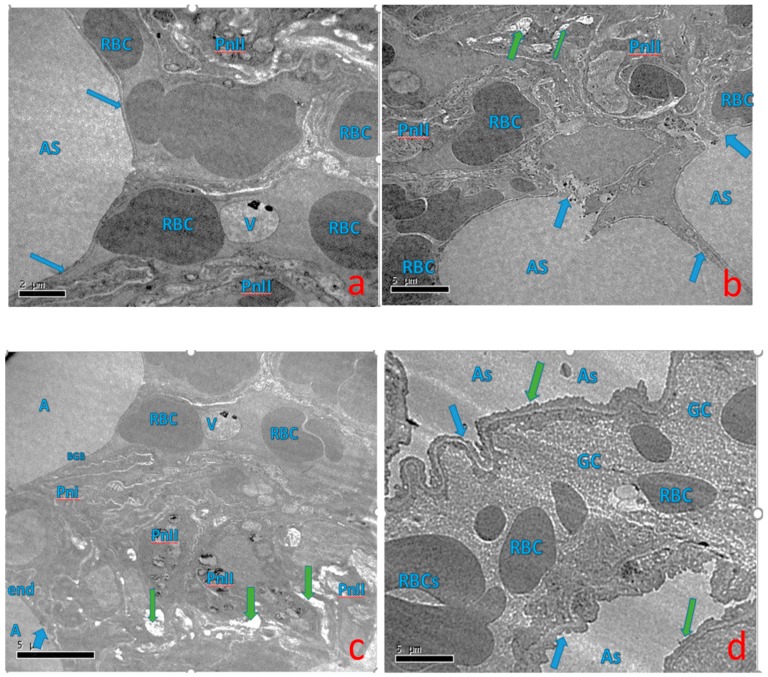
Effects of curcumin treatment on ultrastructural changes in BaP -induced lung tissue: (**a**) control group: lung tissue structure was normal and Normal alveolar space (AS), presence of vacuole (V). There is intact capillary endothelium submerged with alveolar epithelium i.e., normal condition, normal cell membrane of (Type I and Type-II) alveolar epithelial cell (PnI and PnII) and vacuole (V); (**b**) BaP -induced group showed marked irregular and degraded arrangement of microvilli(arrow), the cytoplasmic contents(arrow) escapes into the alveolar lumen, necrotic degenerative changes and ruptured cell membrane of (Type-II) alveolar epithelial cell (PnII). There is a condensed nucleus and dilated vesicles of rough endoplasmic reticulum showing compression of the interalveolar spaces that appears as clefts (green arrow); (**c**) benzopyrene plus curcumin treated group: Less necrotic degenerative changes and normal cell membrane of (Type-II) alveolar epithelial cell (PnII). Normal gas barrier facing alveolar lumen (A); red blood cells are seen in the capillary lumen (RBC). Decreased vacuolization, lung showing compression of the interalveolar spaces which appear as clefts (green arrow); (**d**) curcumin (50 mg/kg bw) group: the alveolar lumen (AS), contains normal red blood cells (RBC), a granulated cytoplasm normal. Regular arrangement of microvilli normalization and Normal Alveolar spaces with intact Blood gas barriers (arrow) and presence of less RBCs.

The present study observed and analyzed the ultrastructural changes of the rat lungs by transmission electron microscopy. In the control group, lung tissue structure was normal and normal alveolar space (AS), and presence of vacuole (V). There is intact capillary endothelium submerged with alveolar epithelium i.e., normal condition, normal cell membrane of (Type I and Type-II) alveolar epithelial cell (PnI & PnII), and vacuole (V). In the BaP treated group, lung epithelial cells showed marked irregular and degraded arrangement of microvilli (arrow), the cytoplasmic contents (arrow) escapes into the alveolar lumen, necrotic degenerative changes, and ruptured cell membrane of (Type-II) alveolar epithelial cell (PnII). There is a condensed nucleus and dilated vesicles of rough endoplasmic reticulum showing compression of the interalveolar spaces that appear as clefts. In the BaP plus curcumin group, it was observed that less necrotic degenerative changes and normal cell membrane of (Type-II) alveolar epithelial cell (PnII). Normal gas barrier facing alveolar lumen (A) and red blood cells are seen in the capillary lumen (RBC). Decreased vacuolization and lung showing compression of the interalveolar spaces which appear as clefts (green arrow) (Figure 5).

### 2.6. Measurement of Apoptotic Bodies

**Figure 6:**
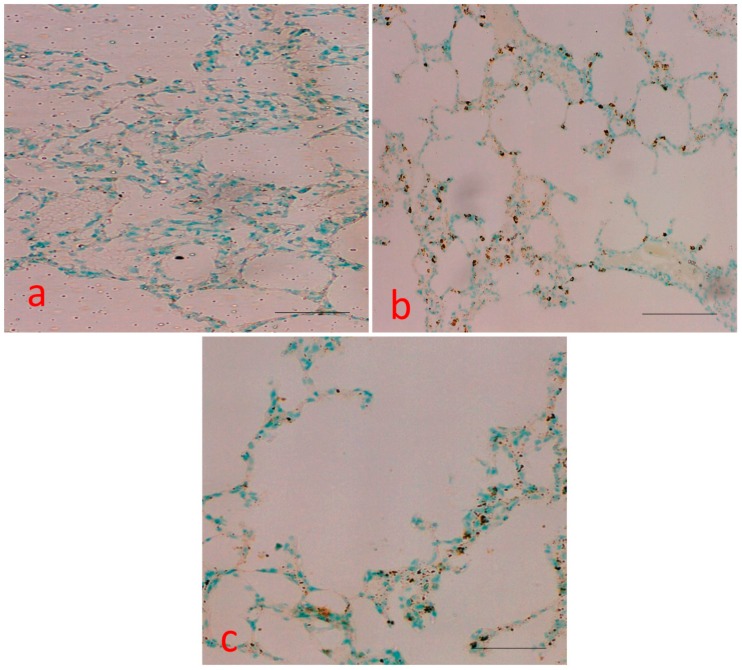
Effect of curcumin treatment on apoptosis in BaP -induced lung tissue: (**a**) control group: did not show apoptotic nuclei; (**b**) TUNEL-positive cells were detected in the BZ group treated cells; (**c**) benzopyrene plus curcumin treated group: curcumin significantly decreased the number of TUNEL-positive cells compared with the number in the BaP group. Scale bar = 50 μm.

In the TUNEL assay, the nuclei of TUNEL-positive (apoptotic) cells were stained brown (Figure 6). The number of apoptotic cells was assessed in all treated groups. Apoptotic activity was quantified by the apoptotic index, which represented the percentage of apoptotic epithelial cells in each tissue. A total of four fields from each section were selected, and cells from each field were counted at a final magnification of 100X. More TUNEL-positive cells were detected in the BaP treated group, whereas control tissue and curcumin treated group did not possess apoptotic nuclei. Curcumin significantly decreased the number of TUNEL-positive cells compared with the number in the BZ group, suggesting a protective effect on BaP-induced cell apoptosis.

### 2.7. Effect of Curcumin on p53 Protein Expression

P53 protein expression was measured in the all treated group, and results were interpreted accordingly. Sections from all treated groups were scored semi-quantitatively under a light microscope for the extent of the immune positivity as 0, less than 5% immune positive cells; 1, <15% immune positive cells; 2, 15–45% immune positive cells; and 3, >45% immunoreactive cells. Furthermore, the amount of staining was counted quantitatively as 0, negative; 1, weak; 2, moderate; and 3, high.

**Figure 7:**
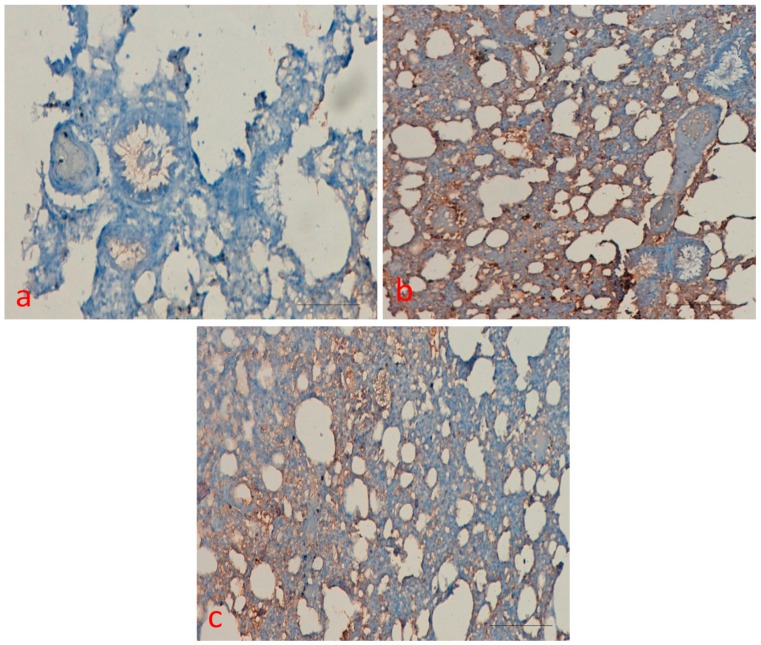
Immunohistochemical analysis. P53 protein expression was evaluated in different treatment groups. (**a**) control group did not show P53 expression; (**b**) expression of p53 was detected in BaP treated group; (**c**) curcumin plus BaP decreased the protein expression as compared with the BaP group and intensity of positivity was low. Scale bar = 50 μm.

As shown in Figure 7, the expression of p53 was detected in the BaP treated group, whereas control and curcumin only group did not show any expression. However, curcumin plus BaP decreased the protein expression as compared with the BaP group and intensity of positivity was low as compared to the BaP only treated group.

### 2.8. Effect of Curcumin on Cell Cycle

**Figure 8:**
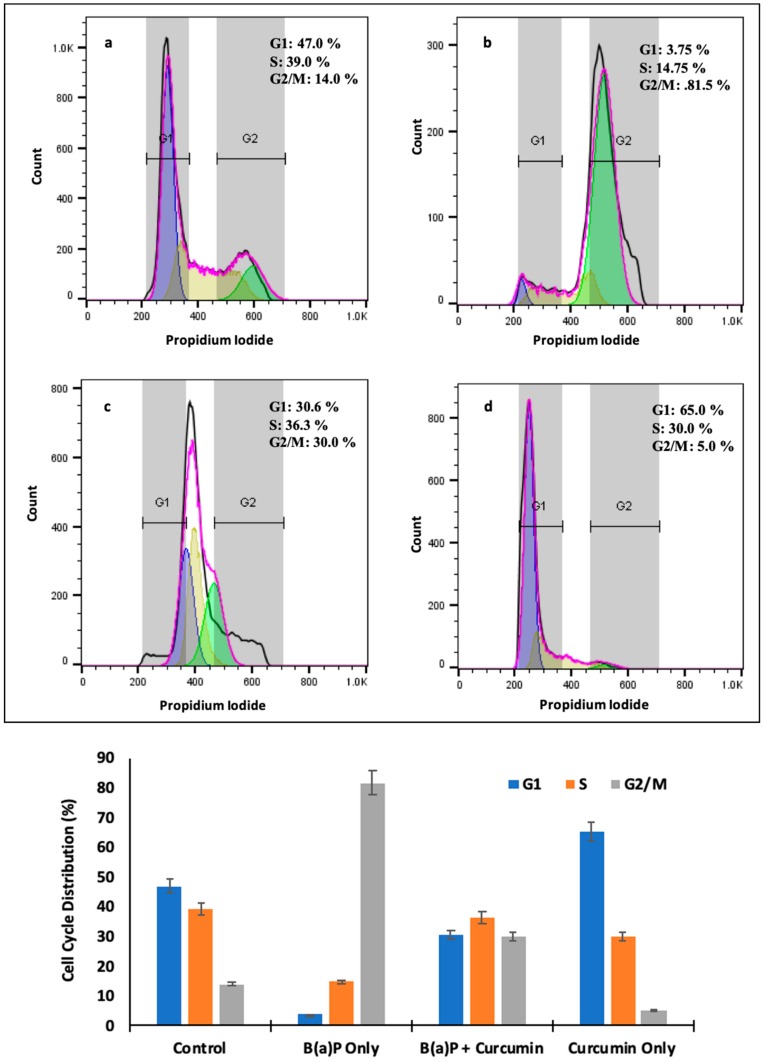
Effect of curcumin on cell cycle phase distribution by flow cytometry (**a**) control group 30.6% cell were arrested in G1; (**b**) high shift in G2/M phase in BaP-only treated group; (**c**) significant change in curcumin + BaP treated group; (**d**) marginal shift was observed from G2/M to G1 in the curcumin only treated group. The cell cycle distributions were calculated and expressed as means ± SDs of three independent experiments.

Flow cytometry data from cell cycle analysis revealed that curcumin significantly decreased the accumulation of cells in G2/M phase by more than 70% as it reached 81.5% in BaP administration alone. It exhibited 30.6% and 36.3% cells were arrested in G1 and S phase, respectively, in the animals treated with curcumin plus BaP, while 3.5% and 14.75% were recorded in BaP only exposed animals. (Figure 8a–d).

### 2.9. Effect of Curcumin on Cell Death

**Figure 9:**
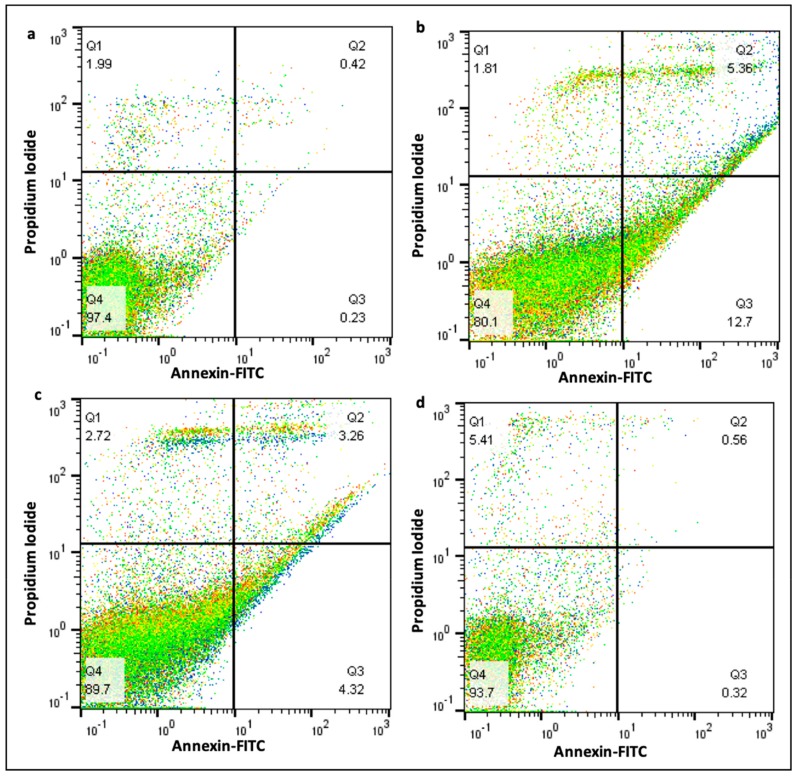

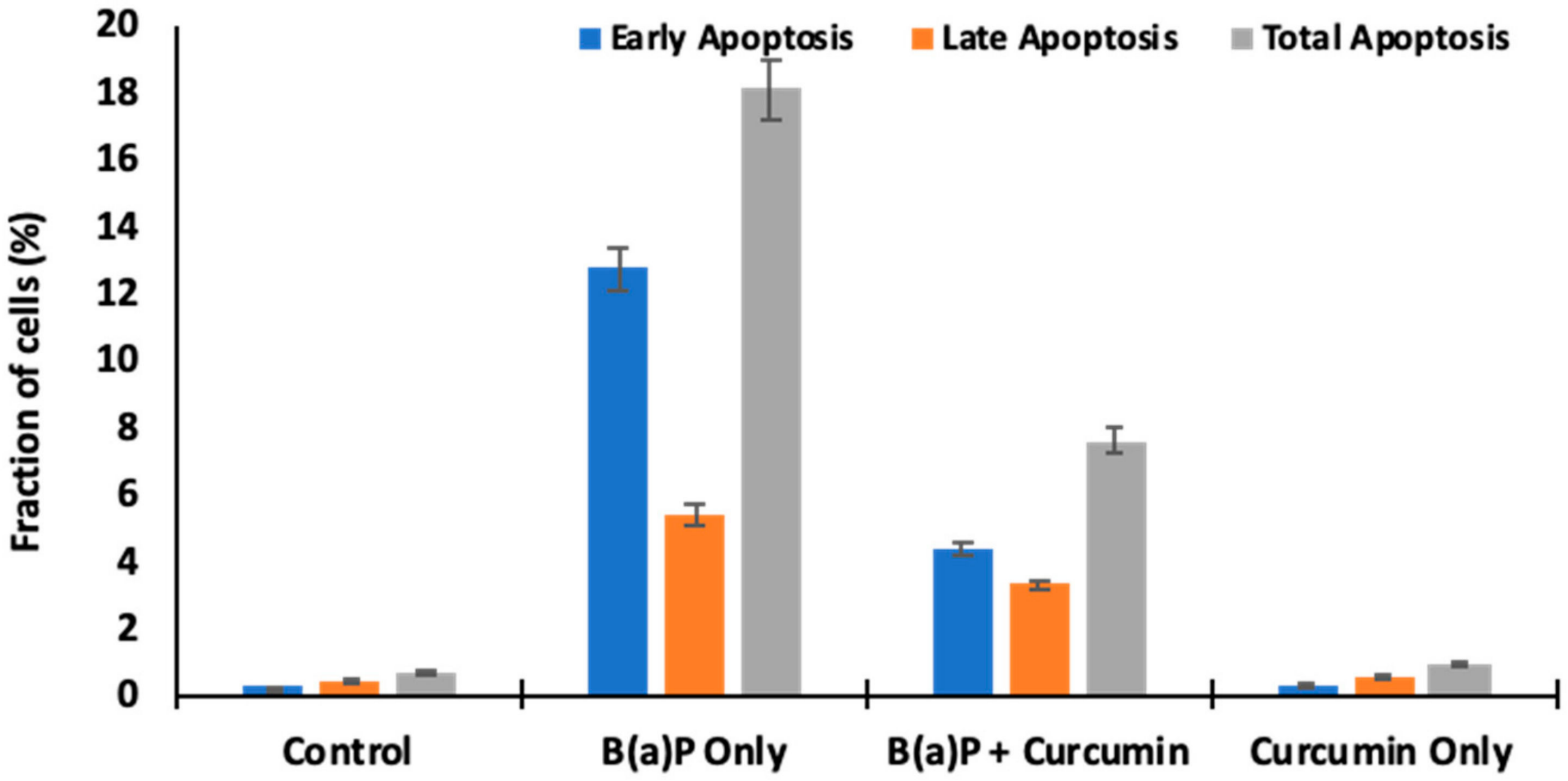
Effect of curcumin on apoptosis by flow cytometry. (**a**) control group 97.4% cells were viable as PI^-^ & Annexin V^−^; (**b**) viable cells decreased to 80 in BaP only group as 12.7% Annexin V^+^ (early apoptosis) and 5.6% PI^+^ and Annexin V+ (late apoptosis); (**c**) curcumin plus BaP group reduced the apoptosis; (**d**) curcumin only group showed no significant difference in comparison to the control group. The cell cycle distributions were calculated and expressed as means ± SDs of three independent experiments.

Flow cytometry data showed that curcumin reduced the apoptotic cell death level increased by BaP. As shown in Figure 9, it was observed that 5.56% late apoptosis and 12.7% early apoptosis in the animals treated with BaP alone. The significant change was found in the animals treated with curcumin and BaP as to 3.26% late apoptosis and 4.32% early apoptosis (Figure 9a–d). Moreover, the total apoptotic events were also reduced to 7.58% by curcumin that raised to 18.06% by BaP (Figure 9c,d)

## 3. Discussion

Various studies have shown that high levels of air pollutants including benzo(a)pyrene are linked with many adverse clinical complications comprising various organ injuries such as lungs, liver, and kidneys with increased mortality as well as morbidity [9,12,18,19]. However, the mechanisms accountable for such association have not been fully explained; few studies showed increased oxidative stress, inflammation, apoptosis, and impairment of cell cycle was due to exposure tobenzopyrene. Previous studies had confirmed that the pathogenesis of lung injury involved in alterations of different cell signaling and metabolic pathways [9,18,20].

This study provides suggestion for the capacity of curcumin to regulate cellular death, inflammation, and antioxidants production in BaP induced lung damage in rats. Notably, our experimental outcomes also provide an evidence for further analysis of curcumin or structurally related analogs as an inhibitor of cytokines and cytokines-regulated genes. The anti-inflammatory tumor suppressing activity and anti-oxidative effects of curcumin have been studied by earlier investigators [21,22,23]. Howbeit, its ability to regulate apoptosis and/or cell cycle is the most vital one. Our aim of this study was to find out the protective effects of curcumin on BaP induced lung damage. Curcumin is a natural antioxidant which can efficiently scavenge free radicals and also be a regulator of antioxidant enzymes in a way mitigating damage during oxidative stress [24,25]. In this study, we reported that exposure of BaP increases inflammatory cytokines, oxidative stress, and apoptosis, which was modulated by the adding of curcumin treatment in rats, and such differences were statistically significant.

Similarly, a recent study also showed a beneficial effect of curcumin on oxidative stress and inflammatory responses regulating HO-1/CO/P38 MAPK expression in outdoor particulate matter (PM2.5) induced lung injury [26].

Instillation of BaP to rats induces lung toxicity, injury, and various other alterations, evidence by histopathological changes specifically interstitial inflammatory cell infiltration, intra-alveolar haemorrhage, intra-alveolar edema, and collagen deposition, which were considered to play a vital role in the pulmonary injury in several earlier studies [10,20,27]. Treatment with curcumin remarkably improved the histopathological parameters in BaP-induced lung injury and lung tissue damage was statically less or mild. The results were in agreement with previous reports that showed curcumin, and its analogs have a protective effect on pulmonary injury in different acute as well as chronic lung injury models [22,28,29]. Lipid peroxidation is one of the leading mechanisms of reactive oxygen species-induced cell damage. Exposure to benzopyrene can lead to lipid peroxidation, an indirect marker of oxidative stress affecting the severity of lung injury. The antioxidant enzymes, such as superoxide dismutase, glutatione peroxidase, and catalase, represent the defence response system to oxidative stress and normalize the adverse effects caused by oxidative stress. Antioxidant enzyme such as superoxide-dismutase catalyzes the dis-mutation of two superoxide anions to hydrogen peroxide and oxygen, and then catalase reduces two hydrogen peroxide molecules to water as well as oxygen [30,31]. In our study, the benzopyrene exposure significantly increased the activity of lipid peroxidation and decreased the activity of SOD, total antioxidant capacity and catalase, while treatment with curcumin enhanced the activities of total antioxidant capacity, GPx, SOD, and catalase which helps reducing oxidative stress. It was demonstrated that overexpression of catalase reduces the levels of benzopyrene metabolites as well as enhances detoxification in endothelial cells [32] and catalase null mice are more sensitive to oxidant tissue injury [33]. Administration of nanoformulated liposome-entrapped superoxide dismutase and catalase into rats play a vital role in the increase of lung-related enzyme specific activities and lung injury [34]. Several clinical and pharmacological investigations have explained antioxidant and anti-inflammatory potential of curcumin in many organ injuries [24].

P53, the tumor suppressor protein, induces apoptosis and cell cycle arrest in different organs injury. Induction of p53 occurs in response to DNA-damaging agents to protect against carcinogenesis. Immunohistochemistry findings demonstrated that p53 levels and TUNEL staining were upregulated following BaP-induced toxicity in rats, which declined or decreased after treatment with curcumin. In addition, transmission electron microscopy results also showed marked irregular and degraded arrangement of microvilli, the cytoplasmic contents escapes into the alveolar lumen, necrotic degenerative changes and ruptured cell membrane of (Type-II) alveolar epithelial cell (PnII). There is a condensed nucleus and dilated vesicles of rough endoplasmic reticulum showing compression of the interalveolar spaces which appears as clefts in the BaP lung injury group, which was significantly improved in the curcumin treatment group. The stress induced by BaP arrested the cell in G2 checkpoint to prevent the cells from entering mitosis. As shown in Figure 6, Figure 7, Figure 8 and Figure 9, the persuasive association between G2/M arrest and the induction of apoptosis was found. A recent study reported that the expression of p53 promotes lung epithelial injury and fibrosis in a bleomycin induced lung injury model [35]. Consistent with our studies, several studies suggested a protective role of curcumin in inhibition of p53 and apoptosis in different pulmonary cell lines and lung injury models [36,37], and curcumin has a potential role to inhibit benzopyrene induced p53 activity in the lung epithelial cell line [38].

However, in contrast to our findings, Liu et al. revealed that deletion of p53 from neutrophils and macrophages attenuated production of pro-inflammatory cytokines and NF-κB activity, which showed protection from LPS-induced lung injury [39]. The stress induced by BaP arrested the cell in G2 checkpoint to prevent the cells from entering mitosis, which was significantly reduced after curcumin treatment. As shown in Figure 6, Figure 7, Figure 8 and Figure 9, the persuasive association between the G2/M arrest and the induction of apoptosis was found. Several parallel studies also showed the effect of BaP induced cell cycle arrest in different cell lines [40,41,42]. Moreover, several studies showed cell cycle inhibitory potential of curcumin and its derivatives in lung cells and different cancer cells [43,44]. Most of the studies on different cells proved that curcumin has apoptotic potential in different cancer cells [45,46]. However, our experiments revealed that BaP- induced cell death was effectively reduced, confirming curcumins anti-apoptotic activity in lung injury. This may be ascribed to the different dose, duration, or different animal model. Further study is warranted to address this question.

## 4. Materials and Methods

### 4.1. Animals and Experimental Protocol

Thirty-two males of 7-weeks-old Sprague–Dawley rats, weighing around 200–250 g, were used in the current study. The rats were hosted in compliance with the international guidelines of laboratory animal care, and the procedures on animals were approved by the Institutional Animal Care and Use Committee of Qassim University. Animals were housed in standard cages and during this time, they had free access to food and water. They were randomly categorized into 4 groups to evaluate the effect of curcumin in the protection of lung injury. Each group containing 8 rats and they were administered for 9 consecutive weeks as group I served as control group and rats received normal saline solution by oral gavage; Groups II diseases control group: BaP was administered orally in corn oil (50 mg/ kg b.wt) thrice a week for 9 consecutive weeks. Group III co-treatment of BaP and curcumin groups, where curcumin (50 mg/ kg b.wt) was administered orally before BaP was administered orally in corn oil (50 mg/kg body weight). Curcumin was administered orally four hours before BaP treatment and such way of curcumin treatment not interrupt a BaP absorption.

Group IV received curcumin thrice a week at a dose level of 50 mg/kg b.wt. All the animals were sacrificed 24 h after last treatment and lung tissues and blood samples were collected to measure the role of curcumin in the prevention of lung injury.

### 4.2. Measurement of Antioxidant Enz

#### Antioxidant enzymes (SOD, CAT, and GPx) and Total Antioxidant Capacity

Blood sample was collected and serum was separated after centrifugation of blood for 12 min at 1500 g and stored at −20 °C until antioxidant analysis. The serum levels of Glutathione peroxidase (GPx), superoxide dismutase (SOD), catalase (CAT), and total Antioxidant Capacity (TAC) were measured according to the manufacturer’s instructions (Abcam, City, UK).

### 4.3. Measurement of Inflammatory Marker (TNF-α, Interleuin-6), and CRP Level

The levels of tumor necrosis factor (TNF)-α, Interleuin-6, C-reactive protein were measured by commercially available ELISA kits according to the manufacturer’s instructions (Abcam, Cambridge, UK). Concentrations were calculated by generating a standard curve using standard proteins and results were interpreted accordingly.

### 4.4. Histopathological Analysis

For histological examination, lung tissues were collected and fixed in 10% formalin (neutral buffered saline) for two days. The lung tissue was embedded in paraffin wax, sectioned into 5-µm slices and stained with Mayer’s haematoxylin and eosin (H&E). Images were captured under light microscope and result were interpreted accordingly. Moreover, Masson’s trichrome staining (Abcam, Cambridge, UK) was performed on lung tissues to detect collagen and photographs were taken under microscopy and results were interpreted accordingly.

The lung injury or alterations were divided based on their severity and scored semi-quantitatively. The scoring system explained as follows: 0: absent (no inflammatory cell infiltrate), 1: mild (mild haemorrhage as well and inflammatory cell infiltrate), 2: moderate (moderate to marked mixed interstitial inflammatory cell infiltrate,), and 3: severe (interstitial inflammatory cell infiltrate and interstitial fibrosis). Moreover, inflammation index determined semiquantitatively based on the presence of inflammatory cells infiltrating using grade scale as no/absent = 0, mild inflammation = 1, moderate inflammation = 2 and severe inflammation= 3 [47].

Masson’s trichrome staining (Abcam, Cambridge, UK) was performed on lung tissues to detect collagen fiber and four randomly selected fields per section were captured for quantification of collagen fiber, collagen fiber around alveolar space were calculated. Masson’s trichrome staining images were captured using a light microscope (Eclipse Ni-E; Nikon Corporation, Tokyo, Japan) under ×100 magnification and results were interpreted accordingly.

### 4.5. Immunohistochemical Staining

Immunohistochemical staining was performed as previously described with little modification [48] to examine the expression pattern of p53. Briefly, formalin fixed paraffin-embedded tissue blocks were cut in 5-µm thick serial sections. The sections were deparaffinised, rehydrated, and rinsed in phosphate buffer saline. The expression of p53 protein was evaluated on paraffin sections using streptavidin-biotin method according to manufacturer’s instructions. Monoclonal antibody used as primary antibodies for p53 to evaluate the expression pattern among different experimental groups. Antigen retrieval was performed in citrate buffer pH 6.0 to unmask the antigen site. Then, slides were incubated with primary antibody overnight, followed by secondary biotinylated antibody for 50 mints. Sections were washed in phosphate buffer saline and then incubated with streptavidin peroxidase for 45 min. To end, Diaminobenzidine (DAB), chromogen was used, section was counterstained with hematoxylin, and results were interpreted under light microscope and photograph was taken. Sections were scored semi-quantitatively under a light microscope for the extent of the immunoreaction as follows: 0, 0% immunoreactive cells; 1, <20% immunoreactive cells; 2, 20–50% immunoreactive cells; and 3, >50% immunoreactive cells. In addition, the intensity of staining was scored semi-quantitatively as 0, negative; 1, weak; 2, intermediate; and 3, strong.

### 4.6. Terminal Deoxynucleotidyl Transferase-Mediated dUTP-Biotin Nick-End Labeling (TUNEL) Assay

TUNEL assay Kit-HRP-DAB (Abcam, Cambridge, UK) allows the recognition of apoptotic nuclei in paraffin-embedded tissue sections. Assay procedure and detection were performed as per manufacturer’s instruction and TUNEL-positive nuclei were counted and photographs were taken under microscopy and results were interpreted accordingly. Apoptotic activity was quantified by the apoptotic index which represented the percentage of apoptotic epithelial cells in each tissue. A total of 4 fields from each section were selected, and cells from each field were counted at a final magnification of 100×.

### 4.7. Transmission Electron Microscopy

Lung tissues were fixed in 2.5% phosphate buffered glutaraldehyde (pH 7.4) at 4 °C for 24 h and 1% osmium tetroxide, and then specimens were dehydrated with grades of ethanol and embedded in epoxy resin. Staining the ultrathin sections by uranyl acetate and lead citrate according to Hayat [49], and photographed with transmission electron microscopy and results were interpreted accordingly.

### 4.8. Cell Cycle Analysis by Flow Cytometry

The blood samples were treated with ice cold RBC lyzed buffer for 10 min with gentle rocking followed by centrifugation at 250× *g* for 10 min. Then, the cells were re-suspended in sample buffer followed by the addition of Ribonuclease (100 g/mL) and incubated at 37 °C for 30 min. Cells were centrifuged at 300× *g* and resuspended in 1 mL of sample buffer containing 50 g/mL propidium iodide (PI) and further incubated for 30 min at 4 °C. The cells were centrifuged, suspended in 200 µL of sample buffer and then analyzed using a MacsQuant flow cytometer (Miltenyi Biotec, Bergisch Gladbach, Germany).

### 4.9. Apoptosis Analysis by Flow Cytometry

Flow cytometry assay was conducted to evaluate the cell apoptosis using FITC/Annexin V Apoptosis detection kit (Miltenyi Biotec, Bergisch Gladbach, Germany), according to the manufacturer’s instructions. Briefly, the cells were harvested in binding buffer following RBC lysis as mentioned above in the previous subsection and incubated with FITC/Annexin V and PI at room temperature in the dark for cell staining. The cells were centrifuged, resuspended in binding buffer and then analyzed using a MacsQuant flow cytometer (Miltenyi Biotec, (Miltenyi Biotec, Bergisch Gladbach, Germany).

### 4.10. Statistical Analysis 

Information from each experimental group were expressed as means ± SEM. Statistical evaluation between different groups was done by using SPSS software. Statistically significant differences were determined using one-way analysis of variance (ANOVA), and *p* < 0.05 was considered to be statistically significant.

## 5. Conclusions

This is the first study demonstrating the therapeutic potential of curcumin and their mechanisms of action in lung pathogenesis after BaP exposure. Curcumin reverse BaP-mediated alterations and may inhibit BaP-induced lung pathogenesis in rats by inactivation pathways of BaP metabolism. Based on the findings, this study revealed that BaP induced pulmonary inflammatory changes were improved after administration of curcumin as evident by less infiltration of inflammatory cells in alveolar space, less deposition of collagen, and oedema. Curcumin attenuates BaP -induced lung injury, probably through inhibiting inflammation, oxidative stress, and apoptosis in lung epithelial cells, and improving cell proliferation. In addition, curcumin administered to BaP induced rats confirmed the improvement in SOD, CAT and GPx, total antioxidant capacity, and oxidative stress biomarker, inhibits inflammatory cytokines TNF-**α** and IL-6 production, and regulates p53. Flow cytometry data showed that curcumin reduced the apoptotic cell death level increased by BaP and cycle analysis findings revealed that curcumin significantly decreased the accumulation of cells in the G2/M phase. Hence, curcumin can be used as a possible potential therapeutic strategy to treat BaP induced lung injury and other pollution associated disorders.
